# Mechanisms of H2pmen-Induced cell death: Necroptosis and apoptosis in MDA cells, necrosis in MCF7 cells

**DOI:** 10.1016/j.heliyon.2024.e40654

**Published:** 2024-11-24

**Authors:** Nosipho Ntanzi, Rene B. Khan, Mthokozisi B. Nxumalo, Hezekiel M. Kumalo

**Affiliations:** Discipline of Medical Biochemistry, School of Laboratory Medicine and Medical Science, College of Health Sciences, University of KwaZulu-Natal, Durban, South Africa

**Keywords:** H2pmen, Apoptosis, Necroptosis, Necrosis, Cancer

## Abstract

Breast cancer is the second leading cause of cancer-related deaths in women around the world. Several cancer therapeutics have already been discovered and are being used to treat breast cancer. However, most of them cause severe side effects. H2pmen, a tetradentate ligand, was used in this study to investigate its cytotoxic effects on growth, viability, and induction of cell death in MCF7 and MDA cells. The cell viability was determined by treating cells with different concentrations of H2pmen. MTT assay was used to obtain an IC_50_, and the cells were then assayed for membrane damage, apoptotic induction, and metabolism. Protein expression of Bax, p53, Bcl2, and xIAP was identified using Western blot analysis. The gene expression of RIPK1, RIPK3, and MKLK was determined using qPCR. In MDA cells, H2pmen increases cytotoxicity, as evidenced by upregulated LDH and JC-10, and enhances apoptosis, indicated by upregulated caspase-3/7 and Bax. In contrast, MCF7 cells exhibit a more stable profile with downregulated LDH and Annexin V Activity. MCF7 cells also show reduced necroptosis and increased necrosis. These findings highlight that H2pmen induces varied cytotoxic effects across MDA and MCF7 cells, with MDA cells exhibiting more pronounced apoptosis and necroptosis alongside complex anti-apoptotic responses.

## 1 Introduction

Breast cancer is the second most common cause of cancer-related deaths in women around the world [1]. In South Africa, the lifetime risk of breast cancer is 1 in 27, making it the most frequent malignancy among women of all races [2]. Different types of treatments are currently available and used to treat breast cancer, such as chemotherapy, targeted drug therapy, and immunotherapy. However, numerous individuals have to deal with debilitating side effects that frequently start before the treatment, worsen with treatment, and persist into survivorship [3]. The need for more efficient management of aversive side effects of cancer treatments has brought clinical attention due to the adoption of increasingly aggressive cancer treatment techniques during the past 20 years [3]. Ligands and metal-containing compounds have received attention as potential anti-cancer therapies due to their toxicity and efficacy in treating diseases [4].

Metal chelators are natural or synthetic compounds that bind to metal with a high affinity. Metal chelators were initially developed to treat metal overload disorders [5]. However, increasing interest has been shown in their potential as chemotherapy agents [5]. Recent research has found that the tetradentate ligand, N,N′-Bis(2-pyridylmethyl)-1,2-ethylenediamine tetrahydrochloride (H2pmen) forms stable complexes with iron [Fe], Chromium [Cr], Copper [Cu (II)] and zinc [Zn (II)]. Since H2pmen is a novel compound, the biochemical effects underlying its mechanism of cytotoxicity on breast cancer cells have not yet been conclusively explored and investigated. However, it is known that a common mechanism of action for ligands is the chelation of intracellular metal, which releases it for redox cycling and causes reactive oxygen species (ROS) production, thus causing cell death [5].

Regulating or terminating the uncontrolled proliferation of cancer cells is one approach to treating cancer. Two main signalling pathways trigger apoptosis cell death: intrinsic and extrinsic [6]. Various extracellular and intracellular stresses, such as chemotherapeutic drugs, activate the intrinsic pathway [[7], [8], [9]]. These initiators eventually increase the permeability of the mitochondria, facilitating the release of pro-apoptotic proteins such as cytochrome *c* [10,11]. Cytochrome *c* release causes the activation of initiator caspase-9, which leads to executioner caspase-3/7 activation, thus causing apoptosis [12].

The extrinsic pathway is activated by binding a pro-apoptotic ligand to a specialised pro-apoptotic membrane receptor, such as death receptors 4 and 5. Once activated, each receptor can individually form the death-inducing signalling complex (DISC) by binding to procaspases-8 and -9 and the adapter Fas-associated death domain (FADD) [[13], [14], [15]]. The DISC formation results in the activation of caspase-8 and -9, which further cleave and activate effector caspases-3 and -7, triggering the execution of apoptosis [16].

There has been increasing evidence that cancer cells may choose to die via necrosis, which is the uncontrolled cell death process that results in the enlargement of organelles, rupture and lysis of the cell's plasma membrane, and leakage of intracellular contents into the surrounding tissue [17]. Necrosis can also serve as a substitute for necroptosis, another programmed mechanism of cell death.

The molecular mechanisms underlying necroptosis involve the formation of the necrosome. The necrosome is a complex composed of receptor-interacting protein kinase 1 (RIPK1), RIPK3 and mixed lineage kinase domain-like protein (MLKL) [18]. Necroptosis is initiated by the death receptor tumour necrosis factor receptor (TNFR), which binds to its ligand and forms a complex that triggers RIPK1 activation. Phosphorylation of RIPK1 recruits and activates RIPK3 [18,19]. RIPK3 phosphorylates MLKL, causing it to oligomerise into a pore-like shape on the cell membrane [20]. This causes cell membrane rupture, which leads to necroptosis. At present, it is believed that necroptosis serves as an alternative option in case apoptosis fails. This study, therefore, will address the cytotoxic effects of H2pmen on growth, viability, and induction of apoptosis, necrosis, and necroptosis on MCF7 and MDA cells.

## 2 Materials and methods

### 2.1 Materials

The MCF7 and MDA cells were purchased from Highveld Biological (Johannesburg (Jhb), South Africa (S.A.)). Cell culture reagents were purchased from Whitehead Scientific (Jhb, SA). Trypan blue, H2pmen, methyl thiazole tetrazolium (MTT) salt, 0.1M phosphate-buffered saline (PBS), bicinchoninic acid (BCA) kit, and β-actin were purchased from Sigma Aldrich (St. Louis, Missouri, United States of America (USA)). Luminometry Promega kits and Cell Signalling Technology (CST) primary and secondary antibodies were obtained from Anatech (Jhb, SA). Unless stated otherwise, western blotting reagents were obtained from Bio-Rad (Hercules, California (C.A.), USA). All other reagents were purchased from Merck (Darmstadt, Germany).

### 2.2 Cell culture and H2pmen preparation

The cells were grown in complete culture media (CCM), which consisted of Eagle's minimum essential medium (EMEM) supplemented with 10 % fetal calf serum, 1 % L-glutamine, and 1 % penicillin-streptomycin–fungizone at 37 °C (^o^C) in a 5 % carbon dioxide (CO_2_) incubator. The 5 mg of H2pmen was dissolved in 10 ml CCM to prepare a 1288 μM stock solution. Cells cultured only in CCM without H2pmen were used as a control.

### 2.3 Cell viability

Cells (15 000/well) were seeded in 96-well plates in triplicate. After 24 h (h), the cells were treated with different concentrations of H2pmen (0, 25, 50, 100, 250, 500, 750, and 1000 μM) for 24 h. After the treatment, media containing H2pmen was carefully removed, and the cells were incubated with 20 μl of MTT salt solution (5 mg/ml in 0.1 M PBS) and CCM (100 μl/well) for 4 h. The MTT salt and CCM solution were removed, and 100 μl dimethyl sulphoxide (DMSO) was added to each well and incubated at 37 °C for 1 h. The absorbance, directly proportional to cell viability, was then measured in each well using a spectrophotometer (SPECTROstar Nano, BMG Labtech, Ortenberg, Germany) at 570 nm with a reference wavelength of 690 nm. At least three independent experiments were conducted to verify the half-maximal inhibitory concentration (IC_50_). An IC_50_ of 100 μM for MCF7 cells and 50 μM for MDA cells were obtained.

### 2.4 Luminometry (CYP 3A4, JC-10, caspases and annexin V)

The luminometry method was used in this study to measure the activity of cytochrome P450 3A4 (CYP3A4) using the P450-Glo™ assay kit (#V8901/2, Promega, Madison, USA) to quantify mitochondrial membrane potential (ΔΨm) using ΔΨm Assay Kit (#MAK159), to detect the activities of caspase-3/7 (#8090), −8 (# 8200), and −9 (#8210) using caspase-Glo assay kits (Promega, Jhb, SA) and to identify apoptotic and necrotic cells using Real-Time-Glo™ Annexin V Apoptosis and Necrosis assay (#JA10111, Promega, Jhb, SA). The confluent flasks of MCF7 and MDA cells were treated with H2pmen IC_50_ concentrations, while the control cells remained untreated and received CCM only. The IC_50_ treated and control cells were then incubated at 37^o^C in a 5 % CO_2_ incubator for 24 h. After 24 h, the cells were removed from the flasks and seeded into a 96-well luminometer plate (20 000 cells/well in 50 μl 0.1 M PBS). The 25 μl of P450-Glo™ was added to each well that represented P450-Glo™ assay, the 20 μl of JC-10 dye was added to each well that represented ΔΨm Assay, 20 μl of caspase-Glo-3/7, −8, and −9 reagents were also added in wells that represented caspase-Glo assay and finally 20 μl of Real-Time-Glo™ Annexin V reagents were added in wells for annexin V Apoptosis and Necrosis assay. The cells were then incubated in the dark for 30 min (min) at room temperature. Thereafter, the luminescence for each assay was detected using a Modulus™ microplate luminometer (Turner Bio-systems, Sunnyvale, California, USA). Data was expressed as relative light units (RLU).

### 2.5 Lactate dehydrogenase (LDH) cytotoxicity detection assay

The Cytotoxicity Detection Kit (#04744926001, Roche, Mannheim, Germany) was used to quantify extracellular LDH levels. The 50 μl of the treatment supernatant was briefly added to a triplicate microtiter plate. After that, 50 μl of the substrate mixture [dye solution (INT/sodium lactate) and catalyst (diaphorase/NAD^+^)] was added to each well, and the plate was incubated for 30 min at 37^o^C. The stop solution (25 μl) was added to each well, and the absorbance was measured at a wavelength of 490 nm using the SPECTROstar Nano spectrophotometer (BMG Labtech, Ortenberg, Germany). Data was expressed as optical density (OD.).

### 2.6 Western blotting

Western blotting was used to determine the protein expression of Bcl-2 (#15071), Bax (#2774), p53 (#48818), and X-linked inhibitor of apoptosis protein xIAP (#14334). The CytoBuster reagent (#71009; Novagen, San Diego, California), supplemented with protease and phosphatase inhibitors (Roche, Germany, 05892791001 and 04906837001, respectively) was used to extract the total protein in treated and untreated cells. The 400 μl of cytoBuster reagent was pipetted into the 75 cm^2^ flasks containing H2pmen treated and untreated cells. The cells were left on ice for 15 min and subsequently centrifuged (10 000×*g*; 4 °C, 10 min) to obtain a crude protein extract. The bicinchoninic acid (BCA) assay was used to determine protein concentration. The bovine serum albumin (BSA) standards (0, 0.2, 0.4, 0.6, 0.8, 1 mg/ml) were prepared and pipetted (12.5 μl) into triplicate wells of a 96-well plate. Thereafter, 100 μl of BCA solution was prepared and added to each well. The plate was then incubated at 37^o^C for 30 min, and absorbance was measured at 562 nm using the SPECTROstar Nano spectrophotometer (BMG Labtech, Ortenberg, Germany).

The samples were then standardised to a 2.0 mg/ml concentration in CytoBuster. The Laemmli buffer [dH_2_O, 0.5M Tris-HCl (pH 6.8), 3 % glycerol, 10 % SDS, 12 % β-mercaptoethanol, 1 % bromophenol blue] was then added to the standardised protein samples in a 1:4 ratio, and the samples were boiled for 5 min at 100 °C. A Bio-Rad compact power supply (Hercules, California, USA) was utilised to separate protein samples using sodium dodecyl sulphate-polyacrylamide gel electrophoresis (SDS-PAGE) (4 % stacking, 7 % resolving) (1 h, 150 V at room temperature).

The separated proteins were electro-transferred to a nitrocellulose membrane (400 mA; 1 h) and then blocked for 2 h in 5 % bovine serum albumin (BSA). Membranes were subsequently immune-probed with the individual primary antibody (1:1000 in 2 % BSA, for 1 h at room temperature and at 4^o^C overnight). Tris-buffered saline containing 0.5 % Tween-20 (TTBS) was used to wash the membranes (10 min, 5 times) before they were exposed to a secondary antibody (1:2500 in 2 % BSA in TTBS, for 2 h at room temperature).

A Clarity Western luminal/enhancer solution and peroxide substrate solution (#1705061, Bio-Rad) were added to each electro-blotted nitrocellulose membrane to form the antigen-antibody complex. The generated signal was detected using the Chemidoc™ Imaging System (Bio-Rad), and protein expression was analysed with Image Lab™ Software (Bio-Rad). Thereafter, the membranes were quenched with 5 % hydrogen peroxide (H_2_O_2_), rinsed with TTBS (10 min, 3 times at room temperature), blocked in 5 % BSA (2 h; at room temperature), and probed with β-actin (ab8226) (Sigma, St Louis, Missouri, USA) in a 1:5000 dilution with 2 % BSA (30 min) for protein normalisation and loading control. Data was expressed as relative band density (RBD).

### 2.7 Quantitative polymerase chain reaction (qPCR)

The gene expression of *RIPK1, RIPK3, and MLKL* was quantified by isolating RNA from H2pmen treated and control cells using Triazol reagent as per the manufacturer's instructions. The Nanodrop2000 spectrophotometer (Thermo Scientific, Waltham, Massachusetts, USA) was then used to quantify the RNA and standardised to 1000 ng/*μL* (ng/μl). RNA was reverse transcribed by reverse transcriptase into copy DNA (cDNA). A reaction volume containing 4 μl RNA template, 2 μl 5X iScript™ reaction mix, 0.5 μl iScript reverse transcriptase, and nuclease-free water was used to synthesise cDNA (iScript™ cDNA Synthesis kit, Bio-Rad; catalog no 107–8890). The reaction was then subjected to 25^o^C for 5 min, 42^o^C for 30 min, 85^o^C for 5 min, and a final hold at 4^o^C to obtain cDNA.

The qPCR was used to determine mRNA expression using SsoAdvanced™ Universal SYBR® Green Supermix (catalog no. #172–5271, Bio-Rad) prepared according to the manufacturer's instructions. The mRNA expressions of *RIPK1, RIPK3*, and *MLKL* were analysed using specific forward and reverse primers (Table 1). Reaction volumes consisting of SYBR green (6.25 μl), forward primer (0.5 μl), reverse primer (0.5 μl), nuclease-free water (3.75 μl), and cDNA template (1000 ng/μl, 1 μl) were prepared. The mRNA expression was compared and normalised to a housekeeping gene, GAPDH. The reaction was subjected to an initial denaturation (95 °C, 4 min). It was followed by 40 cycles of denaturation (95 °C, 15 s), annealing (RIPK1: 56 °C, 40s; RIPK3: 58 °C, 40 s; MLKL: 58 °C, 40 s) and extension (72 °C, 30 s) CFX96 Touch™ Real-Time PCR Detection System (Bio-Rad). The mRNA expression was determined using the Livak method, and data was expressed as relative fold changes (RFC).

**Table 1:** Primer sequences used in qPCR assay.

	Forward Primer	Reverse Primer
RIPK1	5′-AGGTACAGGAGTTTGGTATGGGC-3′	5′-GGTGGTGCCAAGGAGATGTATG-3′
RIPK3	5′-TAGTTTATGAAATGCTGGACCGC-3′	5′-GCCAAGGTGTCAGATGATGTCC-3
MLKL	5′-CTGAGGGAACTGCTGGATAGAG-3′	5′-CGAGGAAACTGGAGCTGCTGAT-3′
GAPDH	5′-TCCCTGAGTGAACGGGAAG-3′	5′-GGAGGAGTGGG1GTCGCTGT-3′

### 2.8 Statistical analysis

All statistical analysis was performed using the GraphPad Prism v 5.0 software (GraphPad Software Inc., La Jolla, CA, USA). The concentration-response-inhibition equation was used to determine IC_50_ for MTT assay. The unpaired student *t*-test was performed with Welch's correction to determine statistical significance for subsequent assays. Data was expressed as mean ± standard deviation (S.D.). The 95 % confidence interval and *p-*value of ≤0.05 indicated that the data was statistically significant.

## 3 Results

### 3.1 Cytotoxicity

#### 3.1.1 MTT assay

**Fig. 1:**
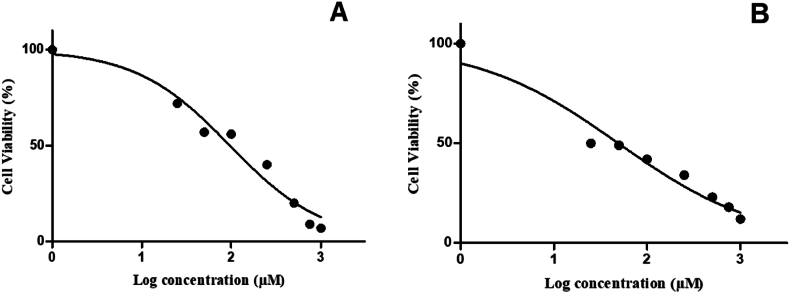
**(A, B):** There was a decrease in cell viability of MCF7 and MDA cells following 24 h treatment with various H2pmen concentrations (∗,∗∗,∗∗∗*p* ≤ 0.05).

The cytotoxicity of H2pmen in MCF7 and MDA cells was determined using the MTT assay. The IC_50_ of 100 μM for MCF7 cells and 50 μM for MDA cells was determined using a dose-response curve generated from serially diluted H2pmen concentrations (0–1000 μM) over a 24 h period. The MCF7 cell viability decreased to 72 % at 25 μM (72.00 ± 2.517, ∗∗*p* = 0.0080) and decreased to about 56 % at 50 μM (56.33 ± 0.8819, ∗∗∗*p* = 0.0004) and 100 μM (55.67 ± 5.925, ∗*p* = 0.0174). The decrease was to less than 50 % at 250 μM (40.00 ± 3.055, ∗∗*p* = 0.0026) and 500 μM (20.33 ± 0.6667, ∗∗∗*p* < 0.0001), and a decrease to less than 10 % was observed at 750 μM (9.667 ± 0.6667, ∗∗∗*p* < 0.0001) and 1000 μM (6.667 ± 0.8819, ∗∗∗*p* < 0.0001) [Fig. 1A].

On the other hand, MDA cell viability decreased to about 50 % at 25 μM (50.33 ± 1.333, ∗∗∗*p* = 0.0007) and 50 μM (49.67 ± 1.453, ∗∗∗*p* = 0.0008). It was slightly above 40 % at 100 μM (42.00 ± 2.082, ∗∗*p* = 0.0013) and slightly above 30 % at 250 μM (33.67 ± 1.453, ∗∗∗*p* = 0.0005). The cell viability was decreased to 23 % at 500 μM (23.00 ± 2.000, ∗∗∗*p* = 0.0007) and further decreased to less than 20 % but more than 10 % at 750 μM (18.67 ± 2.848, ∗∗*p* = 0.0012) and 1000 μM (11.67 ± 0.6667, ∗∗∗*p* < 0.0001) [Fig. 1B].

#### 3.1.2 LDH assay

**Fig. 2:**
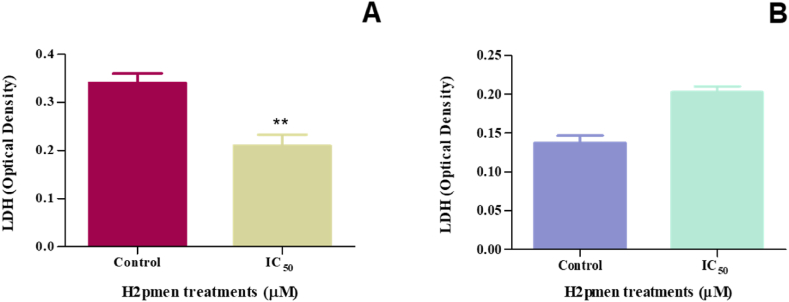
**(A, B):** The H2pmen IC_50_ treatment caused a significant decrease in LDH levels in MCF7 cells while causing an increase in MDA cells (∗∗*p* ≤ 0.05).

The LDH assay was used to measure the cytotoxicity of H2pmen based on measuring the activity of LDH released by MCF7 and MDA cells after H2pmen 24 h treatment. A significant decrease to 0,6-fold in LDH levels was observed at IC_50_ (0,2100 ± 0,01332 O.D., ∗∗*p* = 0.0051) treatment in MCF7 cells compared to the control (0.3403 ± 0.01157 O.D.) [Fig. 2A]. In MDA cells, H2pmen caused an increase to 1,32-fold in LDH at IC_50_ (0,2027 ± 0,004372 O.D., *p* = 0,0796) compared to the control (0,1370 ± 0,007000 O.D.) [Fig. 2B].

### 3.2 Metabolism

**Fig. 3:**
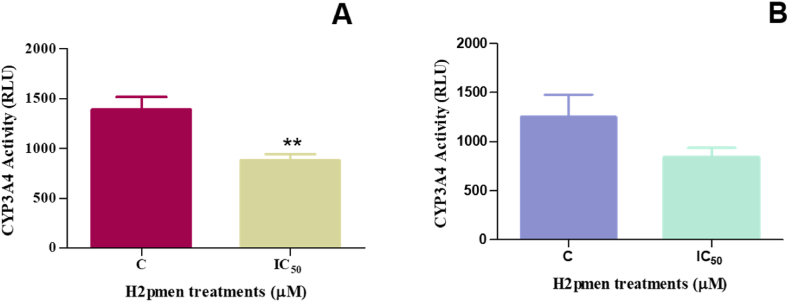
**(A, B):** There was a decrease in CYP3A4 levels in both the MCF7 and MDA cells following 24 h H2pmen treatment (∗∗*p* ≤ 0,05).

The levels of CYP3A4 were quantified in MCF7 and MDA cells to determine if H2pmen was metabolised. There was a significant decrease to 0.66-fold at IC_50_ (926,7 ± 51.98 RLU, *p* = 0,0077) in MCF7 cells relative to the control (1389 ± 50,24) [Fig. 3A] and another decrease to 0,67-fold was noted at IC_50_ (843,4 ± 52.72 RLU, *p* = 0,2482) in MDA cells compared to the control (1251 ± 159,0) [Fig. 3B].

### 3.3 Mitochondrial integrity

**Fig. 4:**
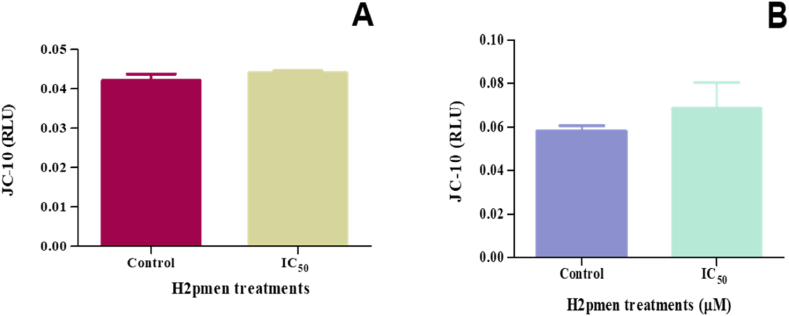
**(A, B)**: There was a slight increase in JC-10 levels at IC_50_ treatment in both the MCF7 and MDA cells.

The mitochondrial membrane potential of MCF7 and MDA cells was assessed using JC-10 assay following H2pmen 24 h treatment. The JC-10 red over blue ratio was used to determine the ΔΨm in cells. There was a slight increase to 1.04-fold in JC-10 levels in MCF7 cells at IC_50_ (0.04417 ± 0.0002667 RLU, *p* = 0.1716) compared to the control (0.04227 ± 0.0008686 RLU) [Fig. 4A]. In MDA cells, JC-10 levels were increased to 1.18-fold at IC_50_ (0.06853 ± 0.006842 RLU, *p* = 0.2743) compared to the control (0.0581 ± 0.001453 RLU) [Fig. 4B].

### 3.4 Apoptosis

#### 3.4.1 Initiator caspases

**Fig. 5:**
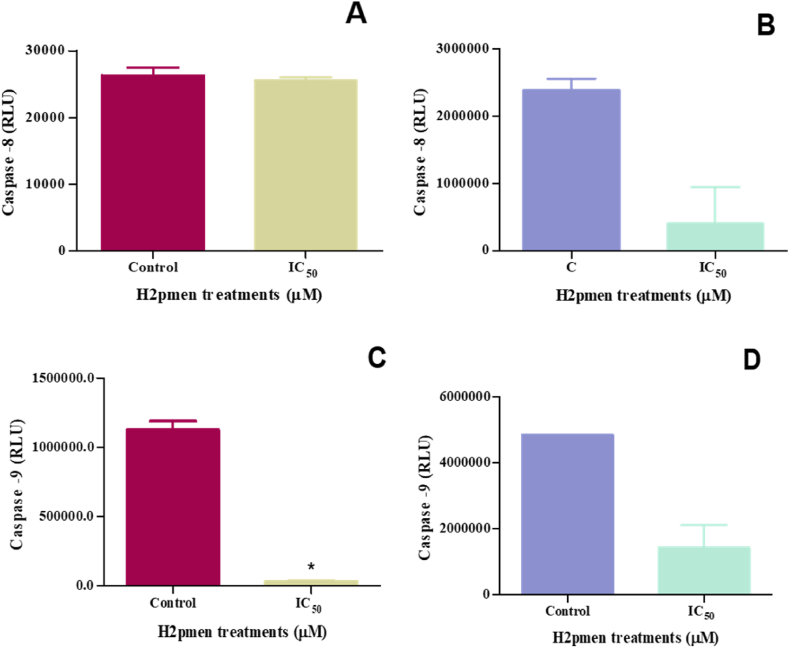
**(A, C)** There was a minimal decrease in caspase-8 and a significant decrease in caspase-9 in MCF7 cells at IC_50_ compared to the controls **(B, D)**: An inconsiderable decline in caspase-8 and -9 was also noted in MDA cells following H2pmen IC_50_ treatments, (∗*p* ≤ 0,05).

To assess if apoptosis was initiated in MCF7 and MDA cells, caspase-8 and -9 activity was quantified using a luminometry assay. Caspase-8 showed a slight decrease to 0,97-fold at IC_50_ (25600 ± 288,7 RLU, *p* = 0,5591) in MCF7 cells compared to the control (26320 ± 817,1 RLU) [Fig. 5A] and a decrease to 0,17-fold decrease at IC_50_ (405700 ± 383200 RLU, *p* = 0,1252) compared to the control (2389000 ± 97260 RLU) in MDA cells [Fig. 5B].

In caspase-9, there was a significant decrease to 0,03-fold at IC_50_ (31700 ± 4629 RLU, *p* = 0,0263) relative to the control (1125000 ± 45000 RLU) in MCF7 cells [Fig. 5C], whereas in MDA cells there was an insignificant decrease to 0,3-fold at IC_50_ (1422000 ± 484100 RLU, *p* = 0,0894) compared to the control (4846000 ± 0,5000 RLU) [Fig. 5D].

#### 3.4.2 Pro-apoptotic proteins (P53 and Bax)

**Fig. 6:**
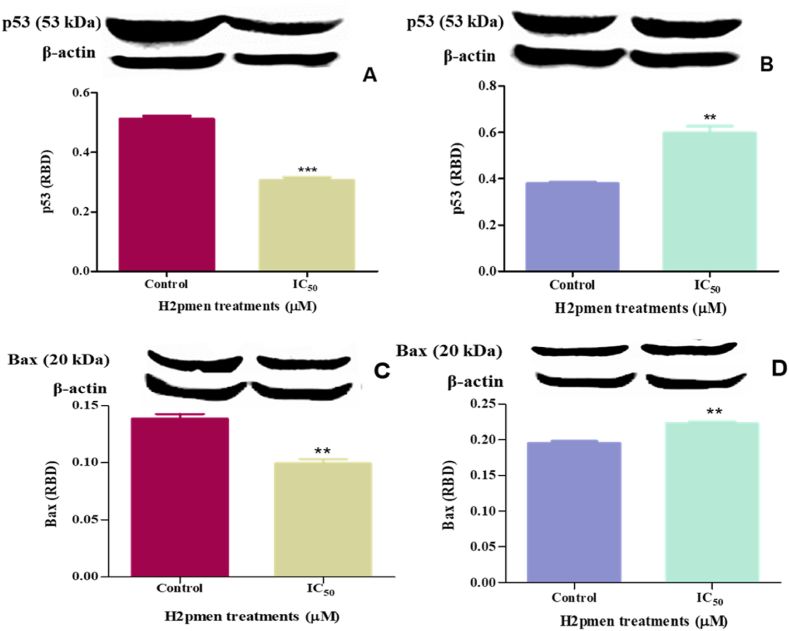
**(A, C)**: The MCF7 cells showed a significant decrease in p53 and Bax protein following 24 h H2pment treatment, (∗, ∗∗∗*p* ≤ 0.05). **(B, D)** In MDA cells, H2pmen significantly increased both Bax and p53 protein expression. (∗, ∗∗*p* ≤ 0.05).

Western Blot was used to quantify p53 and Bax protein expression to ascertain the initiation of apoptosis in MCF7 and MDA cells. In MCF7 cells, H2pmen induced a significant decrease to 0,60-fold in p53 protein expression at IC_50_ (0,3064 ± 0,006073 RBD, *p* = 0,0003) compared to the control (0,5110 ± 0,005937 RBD) [Fig. 6A]. In MDA cells, there was a significant upregulation to 1,58-fold at IC_50_ (0,5992 ± 0,01594, *p* = 0.0055) compared to the control (0,3790 ± 0,003654) [Fig. 6B].

Bax protein expression was significantly decreased to 0,72-fold at IC_50_ (0,09966 ± 0,002096 RBD, *p* = 0,0011) compared to the control (0,1388 ± 0,002314 RBD) in MCF7 cells [Fig. 6C], however in MDA cells it was significantly increased to 1,14-fold at IC_50_ (0,2232 ± 0,001378 RBD, *p* = 0,0010) compared to the control (0,1952 ± 0,001714 RBD) [Fig. 6D].

### 3.5 Apoptosis execution

#### 3.5.1 Caspase-3/7

**Fig. 7:**
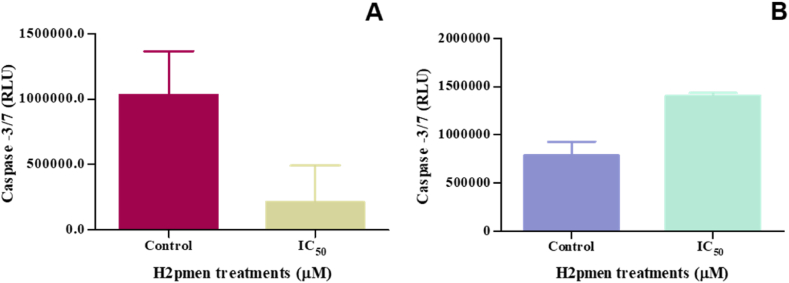
**(A, B)**: H2pmen decreased the activity of caspase −3/7 in MCF7 while increasing it in MDA cells at IC_50_ treatments, (∗*p* ≤ 0,05).

The execution of apoptosis was assessed using caspase-3/7 activity. There was a decrease to 0,21-fold in caspase-3/7 activity at IC_50_ (214300 ± 197000 RLU, *p* = 0,2297) compared to the control (1032000 ± 237300 RLU) in MCF7 cells [Fig. 7A]. However, an increase to 1.78-fold was observed at IC_50_ (1402000 ± 20380 RLU, *p* = 0.1042) in MDA cells compared to the control (1032000 ± 237300 RLU) [Fig. 7B].

#### 3.5.2 Annexin V

**Fig. 8:**
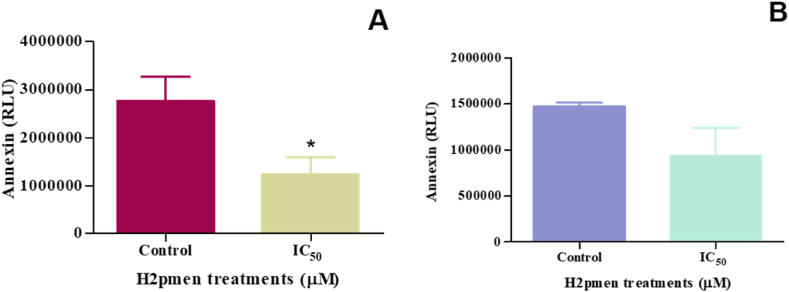
**(A, B)**: There was a decrease in Annexin activity at IC_50_ for both the MCF7 and MDA cells following H2pmen treatment (∗*p* ≤ 0,05).

Annexin V Apoptosis Assay was used to evaluate if apoptosis was executed in MCF and MDA cells. There was a significant decrease to 0,45-fold at IC_50_ (1240000 ± 200100 RLU, *p* = 0,0229) compared to the control (2763000 ± 290400 RLU) in MCF7 cells [Fig. 8A] whereas in MDA cells an insignificant decrease to 0,64-fold was observed at IC_50_ (935400 ± 217400 RLU, *p* = 0,2480) relative to the control (1470000 ± 30000 RLU) [Fig. 8B].

### 3.6 Anti-apoptosis proteins (Bcl2 and xIAP)

**Fig. 9:**
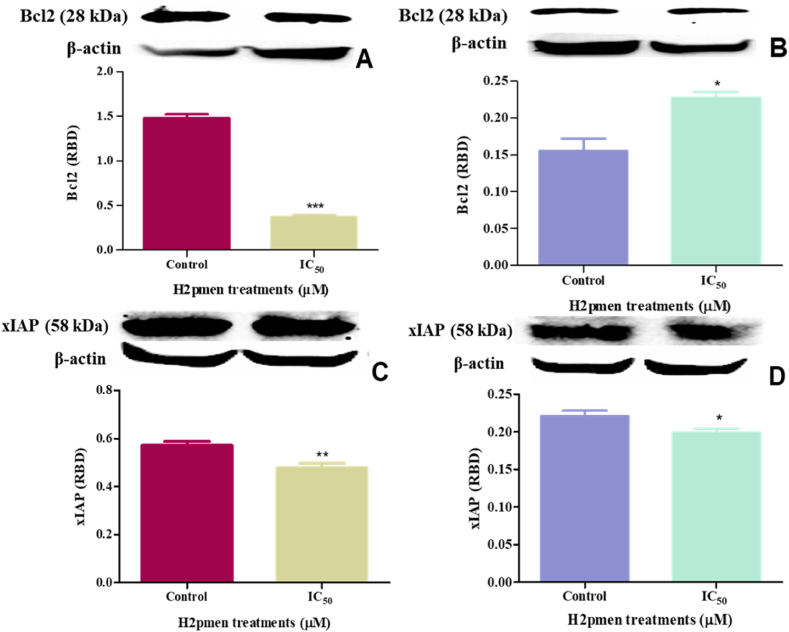
**(A, B)**: There was a significant decrease in Bcl2 protein expression in MCF7 cells and an overexpression in MDA cells (∗, ∗∗∗*p* ≤ 0.05) **(C, D)** xIAP was not expressed in both MCF7 and MDA cells following H2pmen treatments, (∗, ∗∗*p* ≤ 0.05).

Western Blot was also used to quantify the protein expression of Bcl2 and xIAP to assess the inhibition of apoptosis in MCF7 and MDA cells. In MCF7 cells, the Bcl2 protein expression was significantly decreased to 0,40-fold at IC_50_ (0,3712 ± 0,01638 RBD, *p* = 0.0007) relative to the control (1480 ± 0,02365 RBD) [Fig. 9A], while it increased dramatically to 1,63-fold in MDA cells at IC_50_ (0,2275 ± 0,005481 RBD, *p* = 0,0231) treatment relatively to the control (0,1547 ± 0,009830 RBD) [Fig. 9B].

xIAP was significantly downregulated to 0,83-fold at IC_50_ (0,3790 ± 0,003654 RBD, *p* = 0,0070) compared to the control (0,5721 ± 0,009002 RBD)[Fig. 9C] in MCF7 cells and significantly downregulated to 0,90-fold in MDA cells at IC_50_ (0,1992 ± 0,002936 RBD, *p* = 0,0246) relative to the control (0,2208 ± 0,004190 RBD))[Fig. 9D].

### 3.7 Necrosis

#### 3.7.1 Annexin V assay

**Fig. 10:**
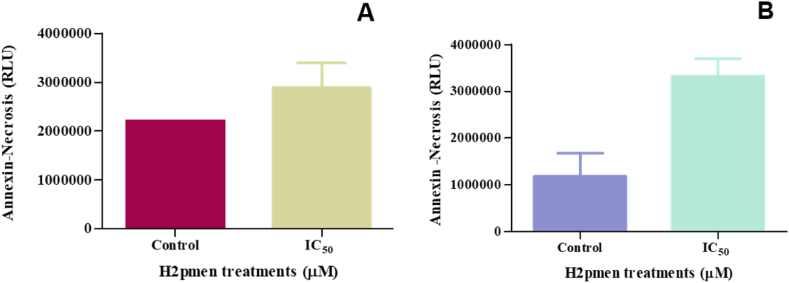
**(A, B)**: An increase in annexin v activity was noted at the IC_50_ in both the MCF7 and MDA cells following H2pmen 24 h treatments, (∗*p* ≤ 0,05).

Annexin V assay was also used to determine if necrosis occurred in MCF7 and MDA cells. In MCF7 cells, the activity of annexin v was increased to 1,83-fold at IC_50_ (2888000 ± 352000 RLU, *p* = 0,3049) compared to the control (2210000 ± 0,5000) [Fig. 10A]. In MDA cells, it was also increased to 4,36-fold at IC_50_ (3325000 ± 265000 RLU, *p* = 0,1280) relative to the control (1183000 ± 347000 RLU) [Fig. 10B].

### 3.8 Necroptosis

**Fig. 11:**
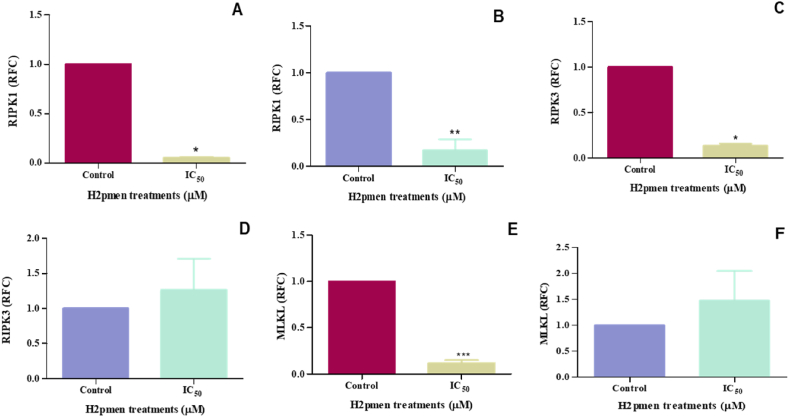
**(A, C, E)**: In MCF7 cells, there was a significant downregulation in RIPK1, RIPK3, and MLKL gene expression, (∗, ∗∗, ∗∗∗*p* ≤ 0.05) **(B, D, F)** RIK1 was significantly downregulated while RIPK3 and MLKL were upregulated in MDA cells, (∗∗*p* ≤ 0.05).

The qPCR method was used to quantify RIPK1, RIPK3, and MLKL gene expression to investigate whether necroptosis was induced in MCF7 and MDA cells. In MCF7 cells, there was a significant decrease to 0,06-fold in RIPK1 expression at IC_50_ (0,0550 ± 0,005000, *p* = 0,0034) compared to the control (1000 ± 0,00003334 RFC) [Fig. 11A], while in MDA cells, there was a significant decrease to 0,22-fold at IC_50_ (0,1700 ± 0,06807, *p* = 0,0067) compared to the control (1000 ± 0,00003334 RFC) [Fig. 11B]. RIPK3 was also significantly decreased to 0,18-fold in MCF7 at IC_50_ (0,1350 ± 0,0150, *p* = 0,0110) related to the control (1000 ± 0,00003334) [Fig. 11C] while it was increased to 1,27-fold in MDA cells at IC_50_ (1265 ± 0,3150, *p* = 0,5548) related to the control (1000 ± 0,00003334) [Fig. 11D]. There was also a significant downregulation to 0,14-fold at IC_50_ (0,1200 ± 0,01732, *p* = 0,0004) in MCF7 cells at IC_50_ compared to the control (1000 ± 0,00003334) [Fig. 11E], in MDA cells, MLKL was upregulated to 1,48-fold at IC_50_ (1480 ± 0,4000, *p* = 0,4423) compared to the control (1000 ± 0,00003334) [Fig. 11F].

## 4 Discussion

Breast cancer remains the most common cancer among women in South Africa. Essential metal ions like iron, copper, and zinc are necessary for cancer cells and healthy cells to grow and proliferate [[21], [22], [23]]. Chelators can target the metabolic pathways of cancerous cells by controlling proteins that regulate these metals and other molecules involved in angiogenesis, cell cycle control, and preventing metastasis [23,24]. Chelation therapy is the preferred medical treatment for reducing the toxic effects of metals. Despite the efficacy of numerous experimental chelators as anti-cancer agents in laboratory settings, only a small fraction have progressed to clinical testing or practical application [25]. H2pmen is a tetradentate ligand that forms stable complexes with iron, Chromium, Copper, and zinc and is an effective reagent for metallic chelation [26]. However, the cytotoxic effects of H2pmen in breast cancer cell lines have not yet been conclusively explored. Therefore, after acute exposure, this study examined whether H2pmen is cytotoxic against MCF7 and MDA cells.

The activation of drugs into reactive metabolites, often dependent on cytochrome (CYP) P450, is the source of many undesirable drug reactions [27,28]. The prominent oxidative enzyme superfamily CYP450 plays a pivotal role in metabolising a wide array of chemotherapeutic drugs and carcinogens. CYP3A4 is the most significant enzyme in this family since it is responsible for the metabolism of most currently available drugs. However, CYP3A4 was inhibited in our study (Fig. 3).

This inhibition can potentially improve the therapeutic efficacy of drugs CYP3A4 predominantly metabolises. In addition, it may lead to increased systemic concentrations of drugs metabolised by this enzyme, thereby enhancing their cytotoxic effects. Consequently, modulation of CYP3A4 activity by the administration of H2pmen represents a promising way to fine-tune the regulation of drug metabolism, with profound implications for therapeutic improvement and toxicity reduction [29].

In this study, cytotoxicity assays were employed to evaluate the impact of H2pmen on MCF and MDA cells. Specifically, the MTT assay was utilised to quantify mitochondrial activity, a proxy for cell viability. This assay converts MTT by viable cells into formazan crystals, measuring cellular metabolic activity and viability [30]. The formazan product is impermeable to the cell membranes, and therefore, it accumulates in healthy cells [30]. This study revealed a dose-dependent decrease in cellular metabolic activity and viability in both MCF7 and MDA cells upon exposure to H2pmen (Fig. 1). This suggests that H2pmen interfered with the cleavage of the tetrazolium ring by succinate dehydrogenase within the mitochondria, a critical step in the MTT assay. This inhibition indicates disruption of the electron transport chain, thereby impairing mitochondrial function. Consequently, the observed decrease in cellular metabolic activity and viability can be attributed to this interference with mitochondrial processes induced by H2pmen.

Reduction in mitochondrial membrane potential (MtMP) is one of the early consequences of the mitochondrial permeability transition in healthy cells. MtMP is a significant indicator of mitochondrial activity since it reflects the mechanisms of electron transport and oxidative phosphorylation, which are the engines behind ATP synthesis. In this study, the JC-10 assay was conducted based on detecting the MtMP changes in cells by the cationic, lipophilic JC-10 dye. The assay revealed a slight increase in the MtMP in MCF7 and MDA cells, which might suggest that the cell's MtMP was not affected by the cytotoxicity of H2pmen. However, Begum (2023) indicated that cancer cells have abnormally high levels of MtMP associated with enhanced invasive properties in vitro. The mechanism underlying the abnormal MtMP in cancer cells is still unknown. In response to cytotoxic exposure, mitochondria can either trigger cell death or activate genes that support cell survival [31]. Cell death frequently results from losing mitochondrial membrane potential, opening channels to remove damaged mitochondria.

Cell viability can be compromised via two primary physiological pathways: actual cell death or disruptions in proliferation attributed to perturbations in cellular metabolism [32,33]. A comprehensive understanding of the precise mechanisms dictating the decline in cell numbers necessitates exploring drug actions [34,35]. A widely used technique to assess cytotoxicity involves measuring the activity of cytoplasmic enzymes released by damaged cells. All cells contain the stable cytoplasmic enzyme lactate dehydrogenase (LDH). Upon disruption of the plasma membrane, LDH is expeditiously liberated into the cell culture supernatant, serving as a biomarker of cellular demise, be it apoptosis, necrosis, necroptosis or other manifestations of cellular damage [35,36]. This understanding offers invaluable insights into the ramifications of drug exposure on cellular viability and the underlying mechanisms dictating such effects. In MCF7 cells, the LDH assay revealed a significant decrease in LDH levels (Fig. 2A), indicating that H2pmen did not induce plasma membrane damage. This suggests that the integrity of the plasma membrane remained intact, resulting in reduced LDH release into the culture medium. Conversely, in MDA cells (Fig. 2B), an increase in LDH levels was observed, implying disruption in membrane assembly and integrity. This discrepancy between the two cell lines suggests differential responses to H2pmen, with MDA cells experiencing membrane damage leading to LDH release, potentially indicating cell death, while MCF7 cells maintain membrane integrity under similar conditions.

There are two possible pathways to initiate cell death. Apoptosis can occur via the intrinsic (mitochondrial) pathway, triggered by internal stress or damage within the cell, or the extrinsic pathway, initiated by external signals from other cells [37]. Caspases are critical regulators of apoptosis; initiator caspases start the apoptosis signal, while executioner caspases perform the bulk proteolysis that triggers apoptosis. This study revealed that initiator caspases-8 (Fig. 5A and B) and −9 (Fig. 5C and D) were downregulated in both MCF7 and MDA cells, suggesting that neither the intrinsic nor the extrinsic apoptosis pathway was activated in these cells. It is known that activated caspases −8 and −9 cleave and activate executioner caspases-3/7. The cleavage and activation of executioner caspases-3/7 result in cellular disruption and cell death [38]. In MCF7 cells, caspase-3/7 was not activated (Fig. 7A), which corresponds with the decreased expression of caspase-8 and -9. This indicates that the apoptotic pathways were not effectively triggered in these cells. However, caspase-3/7 was activated in MDA cells despite the decreased expression of the initiator caspases (Fig. 7B). This suggests that MDA cells might have undergone apoptotic cell death, as the activation of caspase-3/7 is widely considered a critical molecular marker for apoptosis. The unexpected activation of caspase-3/7 in MDA cells may point to alternative apoptotic mechanisms or compensatory pathways that bypass the need for caspase-8 and -9 activation, thus facilitating cell death through different molecular signals.

The apoptotic cell death was further confirmed by the Annexin V assay, which permits measurements of the dynamics of apoptotic death concerning the cell cycle and provides the ability to identify early stages of apoptosis before the loss of cell membrane integrity [39]. The data from MCF7 cells demonstrated a significant decrease in phosphatidylserine (PS) externalisation (Fig. 8A), aligning with the downregulation of caspases and reduced LDH levels. This suggests that the early stages of apoptosis were not detected in these cells. The absence of PS externalisation, a hallmark of early apoptosis, further supports the conclusion that the intrinsic and extrinsic pathways were not activated, as evidenced by the lack of caspase-3/7 activation and the downregulation of caspase-8 and -9. In contrast, MDA cells showed an unexpected but non-significant decrease in PS externalisation (Fig. 8B). Despite the activation of caspase-3/7 in these cells, which typically indicates apoptotic cell death, the annexin V assay did not confirm apoptosis due to the lack of significant PS externalisation. This discrepancy suggests that while MDA cells may have activated downstream apoptotic markers, such as caspase-3/7, the early apoptotic signal involving PS externalisation was not prominently detected. This could indicate alternative apoptotic mechanisms or a bypass of early apoptotic events in MDA cells. Caspases are integral components of the apoptotic machinery and are closely linked to pro-apoptotic proteins like p53 and Bax. P53 mediates apoptosis through a linear pathway that includes Bax transactivation, Bax translocation from the cytosol to membranes, cytochrome *c* release from mitochondria, and caspase-9 activation, which is followed by caspase-3/7 activation [[40], [41], [42]]. This study shows that H2pmen caused a significant decrease in p53 activity (Fig. 6A) and Bax protein expression (Fig. 6C) in MCF7 cells, suggesting that the transcription-independent activation of Bax by p53 did not occur. These results further confirm that no apoptosis was induced in MCF7 cells. However, in MDA cells, there was a significant upregulation in p53 protein expression, as observed in Fig. 6B, and a considerable increase in Bax protein expression (Fig. 6D), suggesting that active p53-induced Bax transactivation and translocation from the cytosol to cell membrane which further led to caspase-3/7 upregulation thus inducing apoptosis. Although initiator caspases were not activated, the overexpression of p53, Bax, and caspase −3/7 provides adequate evidence that apoptosis occurred in MDA cells. Several death checkpoints exist for p53-mediated apoptosis inhibition: direct p53 activity inhibition, mitochondrial function regulation by members of the Bcl-2 family, and caspase inhibitors [43].

Caspases are pivotal effectors in the apoptosis pathway, executing cell death by cleaving specific cellular substrates. Inhibitors of apoptosis proteins (IAPs), including XIAP, counteract this process by directly binding to and inhibiting caspases, thus preventing their activation and impeding the execution of apoptosis. Therefore, assessing IAP levels is crucial to determine their potential role in modulating the observed apoptotic outcomes in the above results [37,43,44]. The anti-apoptotic protein XIAP was downregulated in both MCF7 and MDA cells (Fig. 9C and D), suggesting a minimal role in inhibiting apoptosis. Reduced levels of XIAP would typically result in decreased inhibition of caspases, implying that XIAP is not a significant factor in the apoptosis resistance observed in these cells.

Additionally, regulating Bcl-2, another essential anti-apoptotic protein, further supports alternative anti-apoptotic mechanisms. Specifically, Bcl-2 activity was downregulated in MCF7 cells (Fig. 9A) and upregulated in MDA cells (Fig. 9B). This differential regulation of Bcl-2 suggests that the decrease in caspase-9 expression observed in MDA cells may be attributed to Bcl-2-mediated inhibition.

Inhibition of specific proteins involved in apoptosis regulation in MCF7 cells may have led to a shift in cell death mechanisms towards necrosis. Necrosis is typically an uncontrolled form of cell death, distinct from the regulated processes of apoptosis and autophagy [6]. Necrotic cells were stained using the Annexin V binding assay, and propidium iodide (P.I.) was used to co-stain necrotic and apoptotic cells to differentiate between them since P.I. enters necrotic cells but is not present in apoptotic cells [45]. This study indicated increased P.I. dye binding in the MCF7 and MDA cells in H2pmen-treated cells (Fig. 10), suggesting that H2pmen induced necrotic cell death in both cell lines. Necroptosis, a controlled type of cell death resembling apoptosis and necrosis, is another form of cell death investigated in this work. If caspase-8 is inhibited, RIPK1 interacts with RIPK3 to facilitate MLKL's phosphorylation, which forms a hole in the plasma membrane and induces cell lysis, thus causing necroptosis [46]. This study revealed that there was a significant downregulation in RIPK1(Fig. 11A), RIPK3 (Fig. 11C), and MLKL (Fig. 11E) gene expression in MCF7 cells, which implies that H2pmen did not induce necroptosis in MCF7 cells. However, in MDA cells, there was a downregulation of RIPK1 (Fig. 11B) and upregulation of RIPK3 (Fig. 11D) and MLKL (Fig. 11F) gene expression. The activation of RIPK3 and MLKL suggests that necroptosis might have been induced by H2pmen in MDA cells since RIPK3 and MLKL are known as the most crucial contributors to necroptosis.

Taken together, the MDA cells were very susceptible to the cytotoxicity of H2pmen, but the MCF7 cells exhibited more resistance. The mechanism of resistance induced in MCF7 cells was the downregulation of caspases, pro-apoptotic (Bax and p53), and pro-necroptotic (RIPK1, RIPK3, and MLKL) proteins, resulting in the suppression of apoptotic and necroptotic cell death. Considering that MCF7 is a hormone-responsive cell line with receptors for both estrogen (ER) and progesterone (PR), whereas MDA is a triple-negative cell line lacking ER, PR, and human epidermal growth factor receptor 2 (HER2), the observed differences in response to H2pmen may be due to of these discrepancies in cell lines [47]. Literature has also indicated that estrogen and progesterone enhance the survival of MCF7 cells and facilitate cell cycle progression, hence contributing to treatment resistance [48].

## 5 Conclusion

In conclusion, the analysis of MDA and MFC7 cells highlights their distinct responses to cellular stress. MDA cells are more susceptible to damage, with increased cytotoxicity, apoptosis markers, and heightened necroptosis activity. In contrast, MFC7 cells demonstrate a more stable cellular environment and reduced responses to these stress-induced processes. Future research should focus on understanding the resistance mechanisms in MFC7 cells, potentially leading to targeted therapeutic strategies that exploit the vulnerabilities of MDA cells. Expanding marker analysis to include additional pathways and validating findings in vivo could further inform the development of effective treatments. Additionally, exploring combination therapies targeting apoptotic and necroptotic pathways may enhance treatment efficacy and address diverse cellular responses.

## CRediT authorship contribution statement

**Nosipho Ntanzi:** Writing – review & editing, Writing – original draft, Project administration, Methodology, Investigation, Funding acquisition, Data curation, Conceptualization. **Rene B. Khan:** Validation, Supervision, Resources, Funding acquisition, Conceptualization. **Mthokozisi B. Nxumalo:** Writing – review & editing, Methodology, Formal analysis. **Hezekiel M. Kumalo:** Writing – review & editing, Validation, Supervision, Resources, Funding acquisition, Conceptualization.

## Ethical statement

The study was conducted by using an available cell line. This was an in vitro laboratory study. Hence, no ethical considerations are involved.

## Data availability statement

All data, cell lines, and reagents used in this study are available upon request or commercially available as indicated.

## Funding

This research was funded by the University of KwaZulu Natal 10.13039/501100024216College of Health Sciences and the 10.13039/501100001321National Research Foundation (10.13039/501100001321NRF) of South Africa [grant number: PMDS230510104422] awarded to N.N.

## Declaration of competing interest

The authors declare that they have no known competing financial interests or personal relationships that could have appeared to influence the work reported in this paper.
